# A Review of Nephrotoxicity of Microcystins

**DOI:** 10.3390/toxins12110693

**Published:** 2020-10-31

**Authors:** Shuaishuai Xu, Xiping Yi, Wenya Liu, Chengcheng Zhang, Isaac Yaw Massey, Fei Yang, Li Tian

**Affiliations:** 1 Hunan Provincial Key Laboratory of Clinical Epidemiology, Xiangya School of Public Health, Central South University, Changsha 410078, Hunan, China; xsszll@csu.edu.cn (S.X.); wyl147686@csu.edu.cn (W.L.); zhangcc@csu.edu.cn (C.Z.); mriymassey@csu.edu.cn (I.Y.M.); 2 School of Public Health, Xiangnan University, Chenzhou 423000, China; yixp176911007@csu.edu.cn; 3 Chenzhou Center for Disease Control and Prevention, Chenzhou 423000, China; 4 Hunan Province Key Laboratory of Typical Environmental Pollution and Health Hazards, School of Public Health, University of South China, Hengyang 421001, China; 5 Department of Gastroenterology, Third Xiangya Hospital, Central South University, Changsha 410013, China

**Keywords:** microcystins, nephrotoxicity, phosphatases 2A, oxidative stress, apoptosis

## Abstract

Cyanobacterial blooms triggered by eutrophication and climate change have become a global public health issue. The toxic metabolites microcystins (MCs) generated by cyanobacteria can accumulate in food chain and contaminate water, thus posing a potential threat to human and animals health. Studies have suggested that aside liver, the kidney may be another target organ of MCs intoxication. Therefore, this review provides various evidences on the nephrotoxicity of MCs. The review concludes that nephrotoxicity of MCs may be related to inhibition of protein phosphatases and excessive production of reactive oxygen species, cytoskeleton disruption, endoplasmic reticulum stress, DNA damage and cell apoptosis. To protect human from MCs toxic consequences, this paper also puts forward some directions for further research.

## 1. Introduction

Cyanobacterial blooms owing to global climatologic change and eutrophication have been propagated and widely reported [1,2,3]. The presence of these bacteria may produce a variety of toxins, such as cyanotoxins, which represent potential ecological and public health hazard. Cyanotoxins can be accumulated in aquatic organisms and transferred to higher trophic levels through the food chain [4]. 

**Figure 1:**
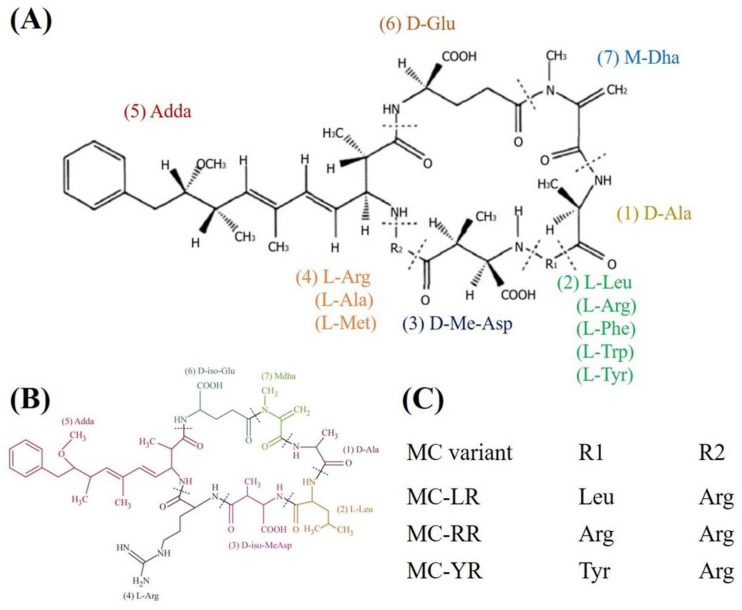
Schematic diagram of the chemical structure of microcystins. (**A**) generic structure of the MCs. (**B**) Microcystin-LR (MC-LR). (**C**) represents some of the most frequent MC congeners (reproduced from [11], 2016, Elsevier Ltd., Amsterdam, The Netherlands).

Among the cyanotoxins, microcystins (MCs) are the most frequently studied due to their wide distribution and high toxicity. MCs are monocyclic heptapeptide hepatotoxins, which basically consist of seven amino acids with a unique side chain of amino acids. The general structure of MCs is cyclo-(-D-Ala-L-X-D-MeAsp-L-Z-Adda-D-Glu-Mdha) [5,6] (Figure 1A). Adda represents (3-amino-9-methoxy-2,6,8-trimethyl-10-phenyldeca-4,6-dienoicacid), a special amino acid necessary for the expression of toxin activity. R1 and R2 at position 2 and 4 represent different amino acids, respectively. MCs are chemically stable compounds and resistant to physical and chemical factors including extreme pH, high temperature and sunlight owing to their cyclic structure [4,6,7]. At present, at least 279 MC variants have been isolated and characterized [8]. On the basis of toxicity, microcystin-LR (MC-LR) is the most common and toxic isomer, followed by microcystin-RR (MC-RR) and microcystin-YR (MC-YR) [9,10] (Figure 1B,C). 

Studies have shown that MCs are capable of impacting human health mainly through contaminated water and food, inhalation, body contact, dietary supplements, and hemodialysis [3,4,12,13,14]. In order to minimize the health hazards induced by MCs, the World Health Organization (WHO) emphasizes that MCs content in drinking water should not exceed 1μg/L [15]. Besides, numerous investigators have reported that MCs can be degraded and detoxified by microbial pathways [16,17].

The primary mechanism of MCs toxicity is the inhibition of protein phosphatases (PP1) and protein phosphatases (PP2A) [18]. It is well established that MCs cause animal and human hepatotoxicity [19,20,21], however, there is also substantial evidence that MCs-dependent impacts are found in the intestine [22,23], lung [24,25], heart [3,26], brain [27,28], testis [29], and kidney [30,31]. Particularly, more and more evidences have been generated suggesting kidney as another major target organ of MCs [32,33,34]. MCs can induce nephrotoxic effects including mitochondria dysfunction [35], endoplasmic reticulum (ER) disturbance [36], DNA damage [37], and cell apoptosis [34]. Nevertheless, this might even increase the possibility of malignant kidney lesions [38,39,40,41]. The purpose of this review was to summarize available evidence on the nephrotoxicity of mammals and fish after exposure to MCs. Besides, the associated challenges were elucidated and further future research that may contribute to protecting humans from MCs hazards were also put forward.

## 2. Distribution and Metabolism of MCs in the Kidney

### 2.1. Distribution of MCs in the Kidney

In most cases, MCs are not easily diffused through the plasma membrane owing to their water solubility, high molecular weight and complicated structure. However, several studies have shown that MCs could be detected in the kidney of mammals and fish under natural and laboratory conditions [42,43,44,45,46,47,48,49,50,51]. It is well known that due to the specificity of cells, a specific pathway from MCs to renal cells may exist [52]. Renal cells have a transport mechanism similar to that of hepatocytes. MCs can be transported into renal cells by multi-specific organic anion transporting polypeptides (OATPs) [53,54] and may generate negative impacts. Feurstein et al. [55] confirmed that the OATP was necessary for renal epithelial cells to actively uptake MCs. Zhang et al. [56] also established that MCs could accumulate in the kidney. ^125^I-MC-LR was injected into mice by three injection methods including intravenous(i.v.), intraperitoneal(i.p.) and peroral (p.o.). Isotope tracer showed that MC-LR was mainly distributed in the blood, liver and kidney. Interestingly, this result was consistent with a previous finding [45]. Wistar rats injected intravenously with extracted MCs at a dose of 80 μg MC-LR eq/kg bw for 1, 2, 4, 6, 12, and 24 h, indicated higher MCs concentrations in kidney compared with the liver [42]. The evidences suggest the kidney as a specific target organ of MC-LR aside the liver and that the toxin can be excreted directly through the kidney.

However, the distribution of MCs in fish is slightly different. Li et al. [43,44] injected 50 μg MCs extracts into crucian carp intraperitoneally and reported MCs accumulation in the blood, followed by liver and kidney. Interestingly, MC-RR rather than MC-LR was detected in renal tissue, which was consistent with the results of another study conducted by Li et al. [57]. Moreover, the authors speculated that the accumulation of MC-RR in the renal tissue was due to organ specificity for different MC variants. At the same time, content of MC-RR was negatively correlated with content of MC-RR in blood, indicating that blood might play an important role in the transport of MC-RR to renal excretion. In a recent study, Bi et al. [48] found that the accumulation of MCs in the liver was the highest, followed by the kidney in the market crucian carp. Jia et al. [49] noted that the MCs content in fish varied in different areas of Lake Taihu, China, and the toxins were mainly concentrated in the digestive organs such as liver and kidney when the bioaccumulation of MCs in four species of fish was assessed. The findings suggest that different species and tissues of fish may react differently to the accumulation degree of MCs. Similarly, Singh et al [50] demonstrated that species differences played a key role in the variation of MCs concentration in the digestive organs (liver and kidney) of common carp and catfish. Further research revealed that MC-LR was located at the nucleus of renal cortex. However, after exposure to 400 μg MC-LR eq/kg bw (MC-LR eq, MC-LR equivalent; bw, body weight) for 1 h in carp, the intracellular localization of MC-LR was in the top part of renal proximal tubular epithelial cells and increased with time [58]. It is worth-knowing that various other studies have also confirmed the presence of MCs in the kidney of swine [59,60] and canines [61,62,63]. Generally, the existing evidence shows that after exposure to MCs, besides the liver, higher MCs content may be detected in the kidney. Thus, the kidney is another target organ for specific distribution of MCs. 

### 2.2. Metabolism of MCs in the Kidney

Glutathione (GSH) is an endogenous substance of detoxification metabolism, which plays a key role in the detoxification of MCs in both mammals and aquatic organism. Hermansky et al. [64] reported that GSH pretreatment could reduce toxic impacts of MCs in mice. Kondo et al. [65] confirmed the presence of microcystins-glutathione (MC-LR-GSH) and microcystins-cysteine conjugates (MC-LR-Cys) in the livers of mice and rats. Many studies have demonstrated alteration of MCs and their conjugates with MCs-GSH after exposure of MCs. Wu et al. [66] studied the metabolic distribution and accumulation of MC-RR-GSH/Cys in carp from Taihu Lake and revealed that the average value of MC-RR-Cys in the carp kidney was four times higher than that in liver. The ratio of MC-RR-Cys/MC-RR in liver/kidney was as high as 5.3/39.8, while the accumulation of MC-RR in kidney was lower, and the formation efficiency of MC-RR-Cys in kidney was higher than that in liver, which indicated that MC-RR-Cys accumulated significantly with the depletion of MC-RR, and selectively excreted further in the kidney. Further research carried out by the same team [67] showed higher MC-LR concentration in kidney of carp. However, the MC-LR-Cys/MC-LR ratio was higher in carp liver, and concentration of MC-LR-Cys in the liver was significantly correlated with MC-LR concentration, indicating that the liver was more active in detoxifying MC-LR through the formation of MC-LR-Cys. The authors believed that there may be a balance between MC-LR-Cys accumulation and purification/metabolism in the kidney. Besides, MC-LR-Cys could be formed directly in the kidney, or transported from the liver or other tissues, and eventually the kidney may be most related to dissociation or excretion of MC-LR. He et al. [68] also found that MCs-GSH conjugate was specific to kidney in herbivorous silver carp, and the kidney could effectively metabolize MCs into its cysteine conjugate. Interestingly, similar findings were observed in rat acute experiments reported by Chen et al. [69]. 

However, although quantitative analysis of these two conjugates, MCs-GSH and MCs-Cys, have been carried out in previous studies, the reason why the content of MCs-GSH in various animal organs was much lower than that of MCs-Cys is still unclear. Further studies are recommended to clarify this. He et al. [68] found that MC-LR and MC-RR were mainly excreted in the form of MC-LR/RR-Cys rather than MC-LR/RR-GSH, indicating that MCs-GSH may be rapidly transformed into more stable MCs-Cys as an intermediate metabolite. Chen et al. [69] and Li et al. [70] also believed that detoxification process of MCs in the kidney was as follows: The metabolite MCs-GSH produced by the GSH binding of MCs in the liver / kidney was transported to the kidney via blood circulation, and MC-GSH was rapidly converted into the downstream metabolite MC-Cys in the kidney to promote excretion. Further, Li et al. [71] proved that MCs were mainly excreted through urinary system, and MC-Cys was the main form of MCs in urine. It is generally speculated that kidney is the main organ of MCs metabolism, and the cysteine binding of MCs may be an important biochemical mechanism for mammals and fish to resist toxic cyanobacteria.

## 3. Nephrotoxicity Caused by MCs on Mammals

### 3.1. Evidences from Epidemiological Investigation

Epidemiological studies are used to clarify harmfulness of MCs, because it can reflect the direct relationship between human health state and MCs exposure. It will be particularly important to identify the impacts of MCs on humans. Table 1 presents a summary of nephrotoxicity caused by MCs in human population.

Proofs of epidemiological study on nephrotoxicity by MCs are limited. Earlier reports on acute renal damage were recorded in Brazil. Patients developed renal insufficiency after using water containing MCs for hemodialysis [72,73]. With the survey on high prevalence of chronic kidney disease of unknown etiology (CKDu) in the Girandurukotte area of Sri Lanka, Liyanage et al. [74] found that the presence of MCs in well water of this area was the reason behind the high incidence of CKDu. Chen et al. [75] also established a positive correlation between serum MCs and abnormal renal function indicators in fishermen. The authors revealed that blood uric acid (UA), blood urea ammonia (BUN), creatinine (SCr), and other indicators together changed in varying degrees, which suggested MCs involvement in kidney function damage on the fishermen. Additionally, a cross-sectional study revealed the relationship between MC-LR in drinking water and aquatic products and renal function damage. This study highlighted individual daily exposure (EDI) to MC-LR through drinking water, and aquatic products intake was found to be an independent risk factor for renal injury. More specifically, compared with the lowest exposure population, the highest exposure population developed BUN, SCr and abnormal filtration of glomerular rate (eGFR), which were 1.80 (95% CI = 1.34–2.42), 4.58 (95% CI = 2.92–7.21) and 4.41 (95% CI = 2.55–7.63), respectively [31]. The evidence suggests that drinking water and aquatic product intake of MCs may be one of the critical risk factors for renal function damage.

### 3.2. Evidences from in Vivo Studies

#### 3.2.1. Microcystis Extracts (MCEs)

Table 2 shows a summary of nephrotoxicity of microcystins crude extracts in in vivo mammalian studies. Male rats were administered an intraperitoneal injection of *Microcystis aeruginosa* PCC7806 extracts at a dose of 0.5, 1.0 and 2.0 lethal dose 50 (LD50). Decreased albumin levels, increased BUN as well as SCr levels, followed by bilirubinuria, proteinuria and hematuria were manifested. In addition, decreased lactate dehydrogenase (LDHL) and aspartate aminotransferase (GOT) levels indicated that cyanobacteria extracts had potential nephrotoxicity [76]. In another acute study, male Kun-Ming (KM) mice were administered an intraperitoneal injection of *Anabaena* extracts from Dianchi Lake at a dose of 0.25, 0.5 and 1.0 g dry weight/kg bw for 24 h [77]. Mice exposed to crude extracts showed obvious toxic symptoms and died within 10–24 h after injection. Meanwhile, the biochemical indexes changed significantly in a dose-dependent manner, and severe histological damage occurred [77]. More interestingly, Humpage et al. [78] found that a new cyanotoxin (AC0243) could also induce acute damage to the kidney of mice. After sub-chronic exposure for 7 days, histological examination indicated that changes in the renal tubular were epithelial disintegration and fibrous deposition. Moreover, acute exposure within 24 h could cause extensive damage to renal cortex and medulla, and even destroy structure of the kidney.

The influence of MCs on the expression of profile of the protooncogenes (c-fos, c-jun and c-myc) was surveyed in the kidney of male Wistar rats injected intravenously with LD50 of MCEs including 86.7 μg MC-LR eq/kg bw [38]. It is worth pointing out that c-jun is a positive proliferation regulator, which has a positive regulatory effect on cell cycle progression. Nevertheless, c-fos has carcinogenic activity, which is overexpressed in tumor cells. C-myc could encode a transcription factor related to cell proliferation and cancerization. Hence, the overexpression of c-myc, c-jun and c-fos in the kidney were probable to clarify potential carcinogenicity induced by MCs [11]. Similarly, Hao et al. [39] reported the transcriptional alterations of tumor-related gene Stathmin in the kidney of male Wistar rats at a dose of 80 μg MC-LR eq/kg bw.

Xiong et al. [29] confirmed that exposure to MCs would lead to variations in antioxidant enzymes at the transcriptional level. Wistar rats were administered an intravenous injection with 80 μg MC-LR eq/kg bw, and the transcription abundance of antioxidant enzymes was modulated in the kidney. The results suggested an adaptative response to combat oxidative injury, confirming that oxidative stress was involved in the damage induced by MCs. Li et al. [79] also confirmed that the mRNA expression of glutathione S-transferase (GST)was inhibited in kidney of Wistar rats injected with MCE, suggesting that oxidative stress was involved in renal damage induced by MCs.

KM mice were fed with MCE extracted from Dianchi Lake for 1 month, and there was no obvious abnormality in renal anatomical morphology and renal coefficient. However, pathological results showed that the structure of renal tubule was incomplete due to cell exfoliation, and there was inflammatory cell infiltration in the intercellular space. MCs affected its physiological function by destroying the structure of nephron [80]. Hence, chronic low-dose exposure to MCE can induce renal injury. Adamovsky et al. [81] also reported nephrotoxicity caused by sub-chronic exposure to MCs. Rats were fed with cyanobacteria extract containing MCs for 28 days. The results showed that malonaldehyde (MDA) increased significantly in the high dose group, while there was no significant difference in biochemical indexes. Meanwhile, histopathology was dominated by the changes of renal cortical tubule system.

#### 3.2.2. Pure Microcystins (-LR)

Nephrotoxicity of pure microcystin-LR in mammalian studies in vivo are summarized in Table 3. Sprague–Dawley (SD) rats and Balb/c mice were injected intraperitoneally with MC-LR for 24 h and 90 min, respectively [82]. In rats, the weight of kidney increased markedly. Pathological results showed that glomerular capillaries were filled with eosinophilic fibrous material after injection of MC-LR for 9 h, and the moderate vacuolation of proximal tubular epithelium was accompanied by mild tubular dilatation at 18 to 24 h. At the same time, serum analysis showed that alanine aminotransferase (ALT), alkaline phosphatase (ALP), total bilirubin (TBIL), BUN, and SCr increased significantly, respectively. However, no obvious serological and histological alterations were found in mice. The findings were consistent with that of Sun et al. [83]. Moreover, Lowe et al. [84] also reported the sub-chronic renal toxicities of MC-LR on Wistar rats via intraperitoneal injection. An increase in glomerular filtration rate, albuminuria and reactive oxygen species (ROS), along with a decrease in Na^+^ reabsorption was observed. Histological results showed increased fibrosis in interstitial space and collagen deposition. The authors believed that MC-LR was the reason for macroscopic changes found in renal parenchyma and renal physiology of the rats. Another acute experiment [85] indicated a significant increase in the renal index and UA levels after Balb/c mice were injected intraperitoneally with MC-LR at a dose of 22 and 43 μg/kg·bw. Xu et al. [86] also found a significant rise in BUN, SCr and MDA after male KM mice were intraperitoneally injected with MC-LR at a dose of 10 μg/kg bw for 13 days. The finding signifies that exposure to MC-LR may cause oxidative stress and kidney function damage. In contrast, Zhong et al. [87] noted no renal lesions in mice exposed to MC-RR. Interestingly Sun et al. [83] also revealed no abnormal changes in renal histopathology, blood biochemical indicators and urinary electrolytes when male KM mice were intraperitoneally injected with MC-RR at a dose of 35, 70 and 140 μg/kg bw for 60 days. It is therefore speculated that the difference in the expression type or amount of OATP family in kidney tissue may be one of the potential mechanisms leading to the difference in nephrotoxicity between rats and mice.

KM mice were given intraperitoneal injection at 3, 6 and 12 μg/kg bw MC-LR, once a day for 7 days [88]. Comparing with the control group, kidney weight of each treated group decreased significantly, while kidney coefficient considerably increased in the middle and high dose group. Meanwhile, it was observed that a certain dose of MC-LR could induce formation of DNA-protein crosslinking (DPC) and increase protein carbonyl content in mice kidney cells. This indicated the emergence of oxidative and DNA damage after MCs exposure. Gaudin et al. [89] also reported that MC-LR could lead to renal DNA fragmentation in mice. A study conducted by Qin et al. [36] revealed that MC-LR could induce renal cell apoptosis. Male ICR mice were treated with 20 μg/kg bw MC-LR for 21 days by intraperitoneal injection, and MC-LR significantly inhibited mRNA and protein expression of endoplasmic reticulum stress (ERS)-related molecules C/EBP homologous protein (CHOP) and caspase-12, whereas it induced weak up-regulation of B-cell lymphoma/leukemia-2 (Bcl-2) in kidney. Consequently, it was not considered that MC-LR can participate in kidney cell apoptosis induced by MC-LR through ERS pathway. 

Male Wistar rats were injected intraperitoneally with MC-LR at a dose of 100 and 150 μg/kg bw, while control group was only treated with saline. MCs resulted in decreased expression of kidney antioxidant enzymes and up-regulation of lipid peroxidation levels, suggesting that oxidative stress played an important role in the pathogenesis of MC-LR-induced nephrotoxicity [90]. Similarly, KM mice treated with 5 μg/kg MC-LR for 15 days showed a significant decrease in antioxidants including GSH, catalase (CAT, superoxide dismutase (SOD) and glutathione peroxidase (GSH-Px), and an increase in the expression level of MDA [91]. However, oxidative damage caused by MC-LR treatment might be reversible. Mice injected intraperitoneally with 30μg/kg bw MC-LR indicated a decline in BUN levels after 1 hour, but increased significantly at 4 h post-exposure, and then after 8 h post-exposure, thus it basically returned to the control group level [92]. Sedan et al. [93] also found that alteration and recovery of the antioxidant system induced by sub-chronic exposure to MC-LR could decrease the activities of GSH, GPH-Px, SOD, and CAT in mice kidney, while nitrate (NOx) activities were significantly increased. It is worth noting that these indicators returned to the control level after the cleaning period. Generally, the sub-chronic experiment suggested that exposure to MC-LR disrupted the redox homeostasis of the kidney, resulting in a certain degree of damage to the antioxidant enzyme system, yet this change might be reversible.

Chronic low-dose exposure to MC-LR also induces renal injury and results in substantial toxicity to urinary system. MC-LR was orally administered to male mice at 1, 30, 60, 90, and 120 μg/L for 3 and 6 months [30]. In the 3-month group, there was no obvious change in SCr, however, BUN decreased significantly at doses of 90 and 120 μg/L. Pathological changes showed that the middle and high dose group had glomerular dilation, compressed bowman’s space, renal tubule dilatation, and interstitium full of eosinophils. Similar, but more pronounced effects were observed in the 6-month group [30]. Male Wistar rats also demonstrated renal tubules expansion with uniform eosinophilic materials, accompanied by collapsed glomerular and thickened basement membrane following MC-LR intraperitonal injection at a dose of 10 μg/kg for 8 months [94]. In further studies [95], rhodamine-podophyllotoxin labeling showed that there were cytoplasmic aggregation and accumulation of fibrous actin filaments in renal tubular epithelial cells. Terminal deoxynucleotidyl transferase-mediated dUTP nick end labeling (TUNEL) revealed that renal cortex and medulla TUNEL-positive cells increased, and the pathological changes induced by MC-LR were more serious than MC-YR. This indicated that chronic exposure to MCs could cause cytoskeleton disruption, apoptosis and necrosis in the kidney.

### 3.3. Evidences from in Vitro Studies 

Table 4 represents a summary of nephrotoxicity of microcystins in in vivo mammalian studies. In previous research, Khan et al. [96] noted that high concentrations of MC-LR and long incubation times impacted rat kidney epithelial cells (ATCC 1571), and typical nuclear changes of apoptosis were observed in renal cells. Chen et al. [97] also demonstrated that exposure to MC-LR promoted apoptotic process by increasing the activation of the apoptosis cascade caspase-3 and Bax/Bcl-2 ratio, resulting in nephrotoxicity in male rats. In another interesting experiment, Nobre et al. [98] revealed that MC-LR could cause damage to isolated kidney. Isolated rats kidney perfused with MC-LR at a dose of 1 μg/ml for 120 min showed significant increase in urine flow and perfusion pressure, as well as significant decrease in glomerular filtration rate and sodium tubule transport fraction. These data indicated that MCs can directly affect kidney by inhibiting renal function. Dias et al. [99] also found that the Vero-E6 cell line (kidney epithelial cells derived from the African green monkey-Cercopithecus aethiops) can be used as a cell model to evaluate the nephrotoxicity of MCs. In a progressive study [40], the team further uncovered that MC-LR could stimulate the cellular process of Vero-E6 cell line. In MC-LR treated group, the activities of mitogen-activated protein kinases p38, c-Jun N-terminal kinase (JNK) and extracellular signal-regulated kinase 1/2 (ERK1/2) increased in a dose-dependent manner, indicating that the proliferation-promoting effect of MC-LR was related to the activation of ERK1/2 pathway. Alverca et al. [100] and Menezeset et al. [101] also demonstrated that MC-LR can reduce the viability of Vero-E6 and participate in the damage of renal cell cytoskeleton through the ERS pathway, resulting in autophagy, apoptosis or necrosis.

Some scholars have carried out studies on the toxicity of MCs in human kidney cells. Fischer et al. [54] found that low concentration of MCs could translate into human embryonic kidney cells (HEK293) through the OATP pathway and lead to renal lesions. In another study, Li et al. [102] revealed that exposure to MC-LR could induce ceramide production in HEK cells. It is worth-knowing that ceramide is an important second messenger in cells and regulates various cellular mechanisms. In this study, ceramide mediated the up-regulation of phosphatases 2A (PP2A) activity and its regulatory subunit protein in HEK293 cells induced by MC-LR. Further MC-LR reduced the polymerization of actin filaments and led to the contraction of tubulin and vimentin. The findings suggest that ceramide-mediated MC-LR may induce PP2A regulation and cytoskeleton instability. Similar, Fan et al. [41] also believed that MC-LR could inhibit the activity of PP2A in HEK293 cells. MC-LR increased the level of c-myc protein mainly by inhibiting the activity of PP2A, which altered the phosphorylation status of serine 62 on c-myc. In addition, MC-LR enhanced the activity of c-myc promoter, which might be related to tumorigenesis in animals and humans.

↑ = effect increase; ↓ = effect decrease.

## 4. Nephrotoxicity Caused by MCs on Fishes 

### 4.1. Microcystis Extracts (MCEs) 

Fischer et al. [58] treated *carp* with MCE at a dose of 400 μg MC-LR eq/kg bw for 72 h and reported that the toxin was mainly distributed in the apical part of the proximal tubular epithelial cells in kidney. Single renal tubular epithelial cell vacuolation, apoptosis, cell exfoliation, and finally protein casting appeared at the cortex-medulla junction, suggesting that exposure to MCs may lead to the death of carp kidney cells. Molina et al. [103] highlighted that MCs caused changes in the activities of acid phosphatase (ACP) and ALP. The histological findings included compressed bowman’s space, necrotic epithelial cells and renal tubules infiltrated by inflammatory cells. Mitsoura et al. [47] also observed similar pathological changes in *carp*.

In an acute study, Lei et al. [44] found invariable amounts of MCs (about 279–1592 ng/g dry weight) in the kidney of *crucian carp* after intraperitoneal injection of crude MCE containing 200 μg MC-LR equivalent/kg bw for 48h, using liquid chromatography-mass spectrometry (LC-MS). The content of MC-RR in the kidney was negatively correlated with that of MC-RR in blood, suggesting that blood played an important role in the transport of MC-RR to the kidney for excretion. Li et al. [104] conducted an interesting field study by collecting *silver carp* fish samples from natural lakes in different months. LC-MS showed that the concentration of MCs in the kidney reached the highest value in July, which was consistent with the time period of bloom outbreak. The renal ultrastructure recovered and MCs decreased or even disappeared after cyanobacteria bloom. It was observed that CAT and GST in the kidney of *silver carp* were significantly higher than those before and after the bloom. Glutathione content in liver and kidney was also high, indicating that *silver carp* had high resistance to MC exposure. Effective antioxidant defense may be an important mechanism of planktonic fish such as silver carp against toxic cyanobacteria blooms. In a follow-up laboratory study [105], *bighead* fish was intraperitoneally injected with crude MCE containing 200 and 500 μg MC-LR eq/kg bw. The results showed that the sensitive reaction of antioxidant enzymes and higher basic glutathione may be the reason for its strong resistance to MCs. Atencio et al. [106] also proved that MCs indeed caused oxidative damage in fish. *Tenca* were exposed to cyanobacteria at a dose of 5, 11, 25, and 55 μg MC-LR, and activities of CAT and SOD were decreased in a dose-dependent manner, while GSH level did not alter significantly. Pathological lesion on *tenca* kidney progressed in expanded bowman’s space and thickened basement membrane with slight bleeding. Qiu et al. [32] also studied the changes of antioxidants in the kidneys of various fish before and after blooms and found that CAT and GST during water bloom were significantly higher than before and after cyanobacterial bloom. Pathological lesions were renal ultrastructural alterations, including fusion of glomerular epithelial cell foot processes and swelled mitochondria of proximal tubules. The authors further believed that chronic exposure to toxic cyanobacterial blooms on kidney damage may be the first step, followed by liver failure. In the physiological state, compared with the liver, the antioxidant capacity of the kidney was weaker. In addition to the effective accumulation of MCs metabolites, the kidney was more susceptible to chronic exposure to MCs. 

### 4.2. Pure Microcystins

Following *Rainbow*s’ intraperitoneal injection with 400 and 1000 μg/kg MC-LR for 26 h, pathological changes of kidney fish composed of coagulated tubular necrosis and bowman’s space dilatation were noted [107]. Ma et al [108] also demonstrated that exposure to MC-LR significantly down-regulated the transcription level of PP2A in the kidney, after *silver carp* were exposed to 50 and 200 μg/kg bw MC-LR. The study suggested that PP2A may be involved in the nephrotoxicity of MCs in *silver carp*. Prieto et al. [109] exposed *tilapia* to MC-LR at a dose of 120 μg/kg for 7 consecutive days. Compared with the control group, activities of CAT, SOD, GSH-Px, and GR decreased significantly in MC-LR-treated group in the kidney of *tilapia*, suggesting that exposure of MC-LR may destroy the redox dynamic balance of *tilapia* kidney and cause nephrotoxicity. Puerto et al. [110] also observed the alterations of antioxidant enzymes after oral exposure of MCs in *tilapia* and noted that the toxin inhibited the expression of mRNA and proteins on GST and GSH-Px. The above two studies provided evidence that sub-chronic exposure to MC-LR may cause changes in antioxidant and detoxification enzymes, and the expression of GSH-Px and GST genes maybe a good indicator of MC-LR-induced nephrotoxicity in fish.

To better understand the relationship between miRNA and MCs-induced nephrotoxicity in fish, Feng et al. [111] injected MC-LR at a dose of 50 and 200 μg/kg bw for 48 h in *silver carp* and reported that the acute MC-LR exposure changed the expression profile of 7 miRNAs related to signal transduction, apoptosis, cell cycle, and fatty acid metabolism in the kidney. The results indicated that adjustments of miRNAs may be related to the nephrotoxicity of MC-LR. Huang et al. [112] confirmed that oxidative stress and cytoskeleton destruction were involved in apoptosis of MCs-induced Grass carp kidney cells (CIK) cells. CIK cells were exposed to 0, 1, 10, and 100 μg/L MC-LR for 48 h. MC-LR induced CIK cells to produce ROS; up-regulate the expression of MDA; and regulate the activities of antioxidant enzymes such as CAT, SOD and GSH, and these changes were more obvious in higher doses. At the same time, cell cycle analysis demonstrated that 1 and 10 μg/L MC-LR guided cell cycle from G1 phase to S phase, and G2/M phase, while 100 μg/L MC-LR reduced the number of cells in G2/M phase, indicating that MC-LR could significantly induce apoptosis in 100 μg/L groups. Furthermore, the transcriptional changes of cytoskeleton genes (β-actin, LC3a and keratin) and the damage of cytoskeleton structure were observed under laser scanning confocal microscope. Thus, oxidative stress and cytoskeleton destruction may interact with each other and jointly lead to apoptosis and nephrotoxicity. Wang et al [34] also reported that MC-LR induced renal cell apoptosis in female *zebrafish* after oral exposure to the toxin at a dose of 1, 5 and 25 μg/L for 60 days. Narrowing of the renal tubule space, which is full of eosinophils and blood infiltration, was observed in the histological damage. RNA-Seq analysis and TUNEL detection showed that exposure to MC-LR could significantly interfere with renal gene expression and induce renal cell apoptosis respectively. In addition, the negative changes of expression of apoptosis-related genes, proteins and enzyme activity in the kidney of zebra fish indicated that MC-LR can cause the production of ROS, and then induce kidney apoptosis through p53-bcl-2 and caspase-dependent pathway. Table 5 represents a summary of nephrotoxicity of microcystins in fishes.

## 5. Potential Mechanisms of Action

**Figure 2:**
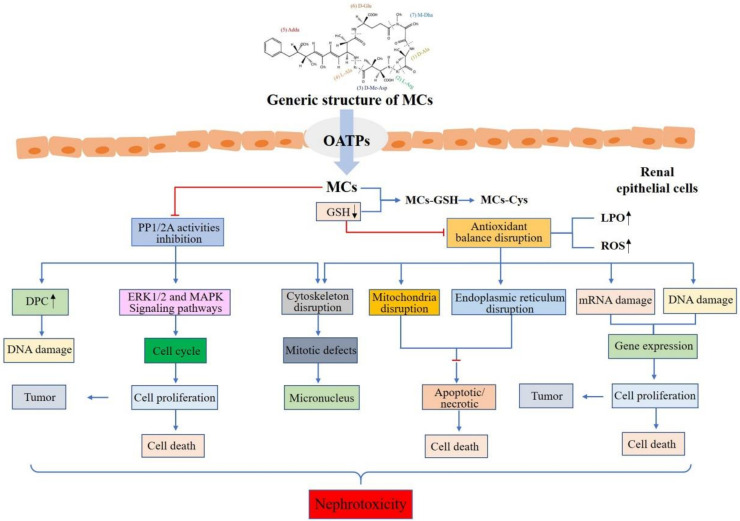
Potential mechanisms of renal toxicity caused by microcystins. (reproduced from [119]).

MCs can not only induce damage to renal cells in vitro, but also induce pathological injury in vivo, leading to the inhibition of protein phosphatases, production of oxidative stress, cytoskeleton disruption, endoplasmic reticulum stress, DNA damage, and cell apoptosis. Figure 2 describes the toxic effect and nephrotoxicity mechanisms of MCs.

### 5.1. Cellular Uptake of MCs

In order to exert nephrotoxicity, a sufficient concentration of MCs must enter the urinary system. Cellular uptake of MCs has been shown to occur entirely through active transport, while passive transmembrane diffusion can be eliminated due to the high molecular weight and structure of MCs [52]. In fact, the active transmembrane transport of MCs is mediated by a specific organic anion transport peptide (OATP) [53,54,113]. Therefore, the systemic distribution of MCs in organs will depend on blood perfusion and the type and expression level of OATP carriers [52,87]. The presence of MCs in the kidney of rodents [42,46] and fishes [48,114] suggests that MCs were able to get across the renal plasma membrane. A meaningful experiment conducted by Sun et al. [83] revealed that after exposure to MC-RR, rats and mice showed different outcomes. In rats group, polyuria and hematuria appeared in the high dose group, and pathological findings were destruction of normal structure of kidney, while serological results showed decreased uric acid and increased creatinine. In contrast, there was no renal damage found in serum biochemical and renal histopathological examination in mice group. Further study found that there were differences in the type and quantity of Oatps expressed in rat and mice kidneys. Oatp1a1 was highly expressed in the kidneys of both rats and mice, while Oatp1a3 was highly expressed in the kidneys of rats. Hence, the difference in renal injury effect between rats and mice may be due to the difference in the expression of Oatps in kidney tissue. The uptake mechanisms of MCs by urinary systems need to be further investigated.

### 5.2. Modulation of PP2A Activities

MCs are highly effective and specific inhibitors of protein phosphatase 1/2A (PP1/2A), which can lead to phosphorylation/dephosphorylation imbalance of key control proteins [18,115,116]. MCs are found to interact with protein phosphatases (PPs) catalytic subunit through a two-step reaction including rapid linking and inactivation of the catalytic subunits, followed by an irreversible binding with cysteine. This combined model is why MCs can inhibit the catalytic activity of PPs and break the balance between protein phosphorylation and dephosphorylation [117]. Recent reports indicated the inhibition of PPs enzyme activity in kidney through both direct and indirect methods [41,102,108,118]. In addition, Dias et al. [40] showed that nephrotoxicity in members of mitogen-activated protein kinases p38, JNK and ERK1/2 of mitogen-activated protein kinase (MAPK) signaling pathways increased significantly under MC-LR exposure. Furthermore, impacts of MC-LR on cytoskeletal disruption, metabolic disorder, cell cycle arrest, abnormal cell proliferation, and cell death have been related to PPs activity and the increased phosphorylation of certain proteins [119].

### 5.3. Oxidative Stress

It is well known that the urinary system is highly sensitive to oxidative stress and lipid peroxidation (LPO), however, their significant increase may lead to renal toxicity [120]. Kidneys are rich in unsaturated lipids, thus they are vulnerable to peroxidation damage. On the one hand, protein carbonylation is widely used to evaluate the degree of oxidative damage of proteins in various biological organisms. After mice were injected with MC-LR for 7 days, Dong et al. [88] found that the content of protein carbonyl in kidney was remarkably increased in high-dose MC-LR group, suggesting that MC-LR had direct oxidative damage on kidney protein. On the other hand, alteration of the antioxidant enzyme system is another reliable evidence to exert oxidative stress. After exposure to MC-LR, the levels of antioxidant enzymes including SOD, CAT, GSH-Px, and GR decreased remarkably in Wistar rats kidney [29,90]. In the case of SD rats kidney, the elevated MDA contents as well as declined antioxidant enzymes involving CAT and SOD showed the occurrence of oxidative stress [121]. Similarly, Kim et al. [122] also discovered that some antioxidant enzymes could be disturbed by exposure to MCs in the kidneys of male Wistar rats. Jayaraj et al. [120] observed a downtrend of antioxidant enzymes in mice kidney exposed to MCs. MCs can also induce oxidative stress in fish, meanwhile, vitamin E can effectively remove ROS and exogenous substances and finally protect the kidney from damage successfully [109]. Similarly, Han et al. [91] also discovered that GSH may reach a certain protective effect on kidney by reducing the lipid peroxidation, improving the antioxidant activity and removing oxygen free radicals under exposure to MC-LR in mice. In vitro studies also showed that the oxidative stress by MCs might induce renal toxicity. MC-LR could induce CIK cells to produce expression of ROS and MDA and regulate the activities of antioxidant enzymes involving CAT, SOD and GSH [112]. However, it is not fully understood why and how MCs exposure can lead to excessive ROS formation that culminates in oxidative damage of the urinary system. This requires more research in the future to overcome this challenge.

### 5.4. Cytoskeleton Disruption

MCs exert a toxic role by causing blistering of cell membrane, loss of membrane integrity, cell coagulation, and the formation of apoptotic bodies, which lead to the disintegration of cytoskeleton [123,124]. The cytoskeleton consists of three elements: microfilaments (MFs), microtubules (MTs) and intermediate filaments (IFs) [125]. In renal cells, MCs exposure triggered the disintegration of IFs and MTs, followed by MFs. In addition, actin under the plasma membrane began to gather and condense into rose-like structures, which eventually collapsed into dense perinuclear bundles, and the cytoskeleton around the nucleus gradually collapsed [117]. Milutinovic et al. [95] revealed that sub-chronic treatment of MCs could induce cytoskeletal alterations. Huang et al. [112] also observed aggregation and collapse of MFs and MTs in CIK cells and even loss of some cytoskeleton structure. Moreover, the authors also detected transcriptional changes of cytoskeletal genes: β-actin, lc3a, and keratin. 

### 5.5. DNA Damage

DNA-protein crosslinking (DPC) is a stable covalent compound formed by DNA and protein. As a molecular biomarker of the toxicity of foreign chemicals, DPC has attracted much attention in recent years [88]. DPC has a certain background level in normal cells. If the body is affected by external physical and chemical factors, it can induce excessive DPC, which will affect gene expression and destroy the structure of chromosomes. Xiong et al. [29] noted that the number of DPC increased significantly when MC-LR was exposed to 3 and 6 μg/kg bw, and the authors speculated that MC-LR exposure could cause DNA damage in mice renal cells. On the other hand, Dias et al. [126] found that DNA lesions were detected in renal micronucleus (Vero-E6) with 5 and 20 μM MC-LR, while the increased DNA damage only occurred temporarily 30 min after exposure to MC-LR by comet assay. In contrast, Gaudin et al. [89] detected that DNA damage in any case would be induced by MC-LR regardless of the route of administration in the kidney. If these increasing lesions occur in kidney, the progress of proliferation in normal renal cell would be affected, resulting in increasing the potential of renal tumorigenesis. Li et al. [38] showed that the expression of c-fos, c-jun and c-myc in kidney might be one possible mechanism for the tumor-promoting activity and initiating activity of MCs. Hao et al. [39] also found that tumor-associated stathmin was significantly correlated with the kidney concentration of MCs. Thus, the alteration of DNA or tumor-associated genes may provide a possible clue to the tumor-promoting potential of MCs in the kidney. 

### 5.6. Apoptosis

There are strong evidences suggesting apoptosis can play a key role in MC-induced renal toxicity [95,112]. Chen et al. [97] detected that the expression of Bax protein increased in different exposure times when rat kidney cells were exposed to different concentrations of MC-LR. Pathmalal et al. [127] investigated the cytotoxicity and possible apoptotic influence of MC-LR on human embryonic kidney cell line (HEK-293) and human kidney adenocarcinoma cell line (ACHN). After exposure to MC-LR for 24 h, the activities of caspase3 and caspase9 in ACHN and HEK-293 cells increased significantly, indicating that the MC-LR induced cytotoxicity and an obvious apoptosis in both ACHN and HEK-293 kidney cell lines. Following exposure of *M. aeruginosa* equivalent to 400 μg/kg MC-LR over 72 h on carp, Fischer et al. [58] observed cavitation for single epithelial cells, cell shedding, apoptosis, and eventual protein-like exfoliation at the cortex-medulla junction. In addition, Wang et al. [34] showed that MCs exposure induced renal cell apoptosis by TUNEL. Additionally, the toxin caused dysfunction in protein processing, cell cycle and oxidative phosphorylation of endoplasmic reticulum. Moreover, Qin et al. [36] explored whether effects of MC-LR on apoptosis of renal cells were through the ERS pathway. The authors found that MC-LR inhibited expression of CHOP and caspase-12 in the kidney, followed by up-regulation of Bcl-2 mRNA, which suggests that apoptosis of renal cells induced by MC-LR may be through the ERS pathway.

## 6. Conclusions

MCs have become the most widely distributed and studied cyanotoxin, and it has become one of the biggest public health problems in the world. Long-term exposure to MCs is prone to cause nephrotoxicity. In the present review, the toxic effects of MCs on kidney were summarized. MCs can cross the glomerular filtration barrier by OATPs after exposure to multiple channels, which may lead to structural and functional changes in the kidney through various mechanisms. Exposure to MCs is highlighted to be fundamentally related to destroying PP2A, cytoskeleton and oxidative phosphorylation system; promote the formation of intracellular ROS; and further induce mitochondrial dysfunction and endoplasmic reticulum stress, which lead to miRNA dysfunction and DNA damage, and finally result in apoptosis or genotoxicity.

However, evidence from high-quality epidemiological study and expounded mechanisms of nephrotoxicity are still limited. Consequently, further studies are required to bridge the study gab. The following are suggested future research directions: (1) Even though many studies have been done on MCs, there is lack of consensus. Therefore, standardized animal model method(s) for assessing acute and chronic MCs exposures should be estimated and established to support direct comparisons between different studies and further to establish evidence weights to support risk assessment. (2) The current drinking water quality standards only provide information to control a few limited microcystins, but there is a relative lack of information on the health effects of a large number of other microcystin variants. Research should be strengthened to provide a basis for government officials to formulate laws and regulations. (3) To strengthen the combined effects of MCs and other environmental pollutants such as melamine, heavy metals, phthalates and pesticide residues to the kidney. (4) To develop accessible and advanced biodegradation technique to degrade MCs from cyanobacterial blooms. (5) Long-term exposure to MCs experiments in laboratory and large cohort studies in high risk area are necessary to verify the relationship between MCs exposure and high incidence of kidney disease. (6) To develop some chemical protective agents against nephrotoxicity caused by MCs. All these scientific challenges and practical values need to be addressed step by step.

## Figures and Tables

**Table 1:** Summary of nephrotoxicity of microcystins in population.

Country/Year	Sample Size	Investigated Effects	Cyanotoxins(Detection Method)	Conclusion	References
Brazil/1996	95	Blood biochemical indicators	MCs (ELISA, LC-MS)	Incident— Cyanotoxin poisoning	Hilborn et al. [73]
Brazil/2001	44	Blood biochemical indicators	MCs (ELISA, LC-MS)	Incident— Cyanotoxin poisoning	Soares et al. [72]
China/2005	76	Renal function indicators	MCs (LC-MS)	Epidemiological study— positive association	Chen et al. [75]
China/2013	5493	Renal function indicators	MC-LR (ELISA)	Epidemiological study— positive association	Lin et al. [31]
Sri Lanka/2016	330	Chronic kidney disease of unknown etiology (CKDu)	MCs (LC-MS)	Epidemiological study— inconclusive	Liyanage et al. [74]

**Table 2:** Summary of nephrotoxicity of microcystins crude extracts in mammalian studies in vivo.

Test Organism/System	Exposure	Toxicant	Concentration/Dose	Time Point	Toxic Effects	References
Male rats	I.P.	Microcystis cell extracts	0.5, 1.0, 2.0 LD50	-	BUN↑, SCr↑, LDH↓, GPT↓, followed with hematuria, albuminuria and bilirubinuria	Bhattacharya et al. [76]
Male KM mice	I.P.	Cyanobacterial crude extracts	0.25, 0.5 and 1.0 g/kg	10, 24 h	BUN↑, SCr↑, T-AOC↓, microstructural damage	Pan et al. [77]
Balb/c mice	I.P.	Cyanobacterial crude extracts	180 and 195 mg/mL	2, 4, 24 h	Microstructural damage	Humpage et al. [78]
Male Wistar rats	I.V.	Cyanobacterial crude extracts	86.7 μg MC-LR eq/kg	1, 2, 4, 6, 12, 24 h	mRNA of c-fos ↑, c-jun↑ and c-mys↑; protein of c-fos↑, c-jun↑	Li et al. [38]
Wistar rats	I.V.	Cyanobacterial crude extracts	80 μg MC-LR eq/kg	1, 2, 4, 6, 12, 24 h	Modulation of CAT, Mn-SOD, Cu, Zn-SOD,GR, GPX, γ-GCS transcription	Xiong et al. [29]
Wistar rats	I.V.	Cyanobacterial crude extracts	87 μg MC-LR eq/kg	1, 2, 4, 6, 12, 24 h	Modulation of 14 GSTs transcription	Li et al. [79]
KM mice	I.G.	Cyanobacterial crude extracts	0.1 mL	1 month	Microstructural damage	Yang et al. [80]
Wistar rats	P.O.	Cyanobacterial crude extracts	136 and 928 μg MC-LR eq/kg	28 days	Ultrastructural damage, MDA↑, GR↑ and LPO↑	Adamovsky et al. [81]

I.P. = intraperitoneal; I.V. = intravenous; ↑ = effect increase; ↓ = effect decrease; - = not determined.

**Table 3:** Summary of nephrotoxicity of pure microcystin-LR in mammalian studies in vivo.

Test Organism/System	Exposure	Concentration/Dose	Time Point	Toxic Effects	References
SD rats	I.P.	20, 40, 80, 120, 160, 180, 200, or 400 μg/kg	1, 12, 24 h	BUN↑, SCr↑, ALT↑, AST↑, TBIL↑, followed with ultrastructural damage	Hooser et al. [82]
Wistar rats	I.P.	55 μg/kg	24 h	Glomerular filtration rate↑, albuminuria↑, ROS↑ and Na^+^ reabsorption↓, microstructural and ultrastructural damage	Lowe et al. [84]
Balb/c mice	I.P.	22, 43 μg/kg	0.5, 4 h	Kidney relative weight↑, UA↑	Lei et al. [85]
KM mice	I.P.	10 μg/kg	13 days	ALT↑, AST↑, ALP↑,BUN↑, SCr↑, MDA↑, LPO↑ and microstructural damage	Xu et al. [86]
KM mice	I.P.	3, 6, 12 μg/kg	7 days	Kidney absolute weight↓, Kidney relative weight↑, DNA-protein crosslinking↑ and protein carbonyl↑	Dong et al. [88]
ICR mice	I.P.	20 μg/kg	21 days	Apoptosis, CHOP↓, caspase-12↓, Bcl-2↑	Qin et al. [36]
Wistar rats	I.P.	100, 150 μg/kg	8 h	GSH-Px↓, GR↓, SOD↓, CAT↓, LPO↑	Moreno et al. [90]
KM mice	I.P.	5 μg/kg	15 days	MDA↑, GSH↓, SOD↓, CAT↓	Han et al. [91]
KM mice	I.P.	30 μg/kg	1, 4, 8 h	ALT↑, SOD↑, CAT↑, BUN first↓ then↑, and all the biochemical indicators are reversible	Li et al. [92]
SD rats	I.P.	30 μg/kg	1, 3, 7, 12 h	Microstructural damage	Li et al. [92]
Male mice	I.P.	25μg/kg	1, 2 months	Kidney relative weight↑, GSH↓, GPH-Px↓, SOD↓, CAT↓, NOx↑ and partial of indicators are reversible after one month’s cleaning period	Sedan et al. [93]
C57BL/6 mice	P.O.	1, 30, 60, 90,120 μg/L	3, 6 months	BUN↓, microstructural and ultrastructural damage	Yi et al. [30]
Wistar rats	I.P.	10 μg/L	8 months	Microstructural damage	Milutinović et al. [94]
Wistar rats	I.P.	10 μg/L	8 months	Microstructural damage, apoptosis↑, cytoskeleton disruption↑	Milutinović et al. [95]

I.P. = intraperitoneal; P.O. = peroral; ↑ = effect increase; ↓ = effect decrease.

**Table 4:** Summary of nephrotoxicity of microcystins in mammalian studies in vitro.

Test Organism/System	Toxicant	Concentration/Dose	Time Point	Toxic Effects	References
Rat kidney epithelial cells (ATCC 1571)	MC-LR	13.3 μM	24 h	Apoptosis↑, ultrastructural damage	Khan et al. [96]
Rat NRK cell	MC-LR	10, 100, 1000 nM	4, 6, 7, 8 h	Bax↑	Chen et al. [97]
Isolated rat kidney	MC-LR	1 μg/L	2 h	Urine flow↑, perfusion pressure↑, glomerular filtration rate↑, sodium tubule transport fraction↓, microstructural damage	Nobre et al. [98]
Vero-E6 cell	MC-LR, cyanobacterial crude extracts	1.4–175 nM	24, 48, 72 h	Cell viability↓	Dias et al. [99]
Vero-E6 cell	MC-LR, cyanobacterial crude extracts	5, 50, 500, 5000 nM	24 h	Cell proliferation↑, P38↑, JNK↑, ERK1/2 activity↑	Dias et al. [40]
Vero-E6 cell	MC-LR	1.3, 2.5, 5, 10, 20, 30, 40, 50, 75, 100, 150 μM	24, 48, 72 h	Cell viability↓, autophagy, apoptosis, necrosis, ultrastructural damage	Alverca et al. [100]
Vero-E6 cell	MC-LR	6, 12, 25, 50 μM	24 h	Cell viability↓, autophagy, cytoskeleton disruption↑	Menezes et al. [101]
HEK293	MC-LR, MC-RR, MC-LW, MC- LF	0.01, 0.1, 1, 10, 100, 1000 nM	4 h	Cell viability↓, phosphatase activity↓, modulation of Oatps expression	Fischer et al. [54]
HEK293	MC-LR	2, 10 μM	24 h	Ceramide↑, PP2A activity↑, cytoskeleton disruption↑	Li et al. [102]
HEK293	MC-LR	10 μM	24 h	PP2A activity↓, alteration of c-myc expression	Fan et al. [41]

**Table 5:** Summary of nephrotoxicity of microcystins in fishes.

Test Organism/System	Exposure	Toxicant	Concentration/Dose	Time Point	Toxic Effects	References
**Microcystins crude extracts**						
*Carp*	I.G.	cyanobacterial crude extracts	400 μg/kg MC-LR	3 days	Microstructural damage, apoptosis, necrosis, cell shedding, proteinaceous casts↑ at the cortico-medullary junction	Fischer et al. [58]
*Tilapia*	Immersion	cyanobacterial crude extracts	60.0 μg MC-LR/fish	14, 21 days	Microstructural damage, ALP↑, ACP↑	Molina et al. [103]
*Silver carp*	Immersion	cyanobacterial crude extracts	-	-	CAT↑, GST↑, GSH↑, GPX↑, SOD↑, ultrastructural changes	Li et al. [104]
*Bighead Carp*	I.P.	cyanobacterial crude extracts	200, 400 μg/kg MC-LR	24 h	CAT↑, microstructural damage	Li et al. [105]
*Tenca*	Immersion	cyanobacterial crude extracts	5, 11, 25, 55 μg		CAT↓, SOD↓, ultrastructural damage	Atencio et al. [106]
*Silver carp, Bighead Carp, Carassius auratus, Culter ilishaeformis*	Immersion	cyanobacterial crude extracts	-	-	Microstructural damage, alterations of antioxidant enzymes	Qiu et al. [32]
**Pure microcystins**						
*Oncorhynchus mykiss*	I.P.	MC-LR	400, 1000 μg/kg	16 h	Microstructural damage	Kotak et al. [107]
*Silver carp*	I.V.	MC-LR	50, 200 μg/kg	1, 3, 8, 12 h	Downregulated PP2A-A transcription	Ma et al. [108]
*Tilapia*	Immersion	MC-LR	120 μg/kg	7 days	Dysfunction in redox dynamic balance, CAT↓, SOD↓, GSH-Px↓, GR↓	Prieto et al. [109]
*Tilapia*	Immersion	MC-LR	60 μg MC-LR	21 days	LPO↑, alterations of antioxidant enzymes, modulation of GPx and GST genes transcription	Puerto et al. [110]
*Silver carp*	I.V.	MC-LR	50 and 200 μg/kg	8, 24, 48 h	Modulation of 7 miRNAs transcription	Feng et al. [111]
*Zebrafish*	Immersion	MC-LR	1, 5, 25 μg/L	60 days	Microstructural damage, modulation of genes transcription, apoptosis, ROS↑	Wang et al. [34]
CIK cell	-	MC-LR	1, 10, 100 μg/L	24, 48 h	Cell viability↓, G_2_/M phase arrest, ROS↑, MDA↑, modulation of antioxidant enzymes including CAT and SOD, modulation of cytoskeletal genes (β-actin, lc3a, and keratin) transcription	Huang et al. [112]

I.P. = intraperitoneal; I.V. = intravenous; I.G. = intragastrical; ↑ = effect increase; ↓ = effect decrease; - = not determined.
